# Emerging Concepts for the Treatment of Biofilm-Associated Bone and Joint Infections with IV Fosfomycin: A Literature Review

**DOI:** 10.3390/microorganisms13050963

**Published:** 2025-04-23

**Authors:** Sara Tedeschi, Efthymia Giannitsioti, Christian Mayer

**Affiliations:** 1 Department of Medical and Surgical Sciences, Alma Mater Studiorum, University of Bologna, 40138 Bologna, Italy; 2 Infectious Diseases Unit, Department of Integrated Infectious Risk Management, IRCCS, Azienda Ospedaliero-Universitaria di Bologna, 40138 Bologna, Italy; 3 1st Department of Propaedeutic and Internal Medicine, Medical School, National and Kapodistrian University of Athens, Laiko General Hospital, 11527 Athens, Greece; gianiemi@hotmail.com; 4 InfectoPharm Arzneimittel und Consilium GmbH, 64646 Heppenheim, Germany; christian.mayer@infectopharm.com

**Keywords:** bone and joint infection, fosfomycin, multidrug-resistant, carbapenem-resistant, *Staphylococcus aureus*, *Klebsiella pneumoniae*, *Pseudomonas aeruginosa*, prosthetic joint infection, diabetic foot infection, biofilm

## Abstract

Due to the involvement of biofilms in the pathogenesis of bone and joint infections (BJI), the treatment of these infections is often challenging, especially when multidrug- or extensively drug-resistant (MDR/XDR) pathogens are involved. Intravenous fosfomycin (FOS) is a phosphoenolpyruvate analogue with a unique mode of action and broad-spectrum activity against both Gram-positive (GP) and Gram-negative (GN) pathogens. It is used in various severe and deep-seated infections, including BJIs. This review article focuses on preclinical and clinical data surrounding the use of FOS for biofilm-related BJIs. Data from several in vitro and animal models of infection demonstrated that FOS, especially in combination with other antibiotics, is effective against biofilms of (methicillin-resistant) *Staphylococcus* spp., (vancomycin-resistant) *Enterococcus* spp., carbapenem-resistant and extended-spectrum beta-lactamase-producing Enterobacterales, and MDR *Pseudomonas aeruginosa*. Data from clinical studies, mostly retrospective observational studies and case reports/case series, revealed that FOS was typically used in combination with other antibiotics for the treatment of various BJI, including acute and chronic osteomyelitis, prosthetic joint infections, and fracture-related infections, in adult and pediatric patients. Success rates often exceeded 80%. FOS exhibits good and fast penetration into bone tissue and is generally well tolerated, with only a few adverse drug reactions, such as gastrointestinal disorders and electrolyte imbalances. Collectively, the data indicate that FOS is a valuable option as part of combination regimens for the treatment of BJIs caused by both GP and GN bacteria.

## 1. Introduction

### 1.1. Biofilm-Associated Bone and Joint Infections

The term “biofilm” was first introduced by Bill Costerton, who described an organized structure of polymeric matrix produced by microbes adhering to the surface of an implant and being furthermore encapsulated into the matrix [1]. The most frequently isolated bacteria in a biofilm are staphylococci (i.e., *Staphylococcus aureus* and *S. epidermidis)*, *Enterococcus faecalis*, *Pseudomonas aeruginosa*, *Escherichia coli*, *Klebsiella pneumoniae*, *Proteus mirabilis*, and *Streptococcus viridans* [1,2]. However, *Enterobacter* spp., *Acinetobacter* spp., *and Candida* spp. can also be biofilm producers, enlarging the spectrum of pathogens able to form biofilm into tissues and implantable foreign materials [2].

Bacterial biofilm is initially formed when microbes adhere to the surface of implants or into tissues and produce “slime”. Within days, bacteria aggregate, and mature biofilm grows up, being equipped with multiple and complex structures in order to embed alive microbes and sustain chronic infection [3,4]. Modern molecular imaging technologies have visualized biofilms, revealing complex populations of cells living in a particular ecosystem of a matrix of glycopolysaccharide deposits of slimes along with permeable water channels, which allow for the exchange of energy and nutrients within this structure [2]. Extracellular polymeric substances (EPS) comprising polysaccharides, lipids, nucleic acids, proteins, lipopolysaccharides, and minerals control biofilm function and protect the embedded bacterial communities from environmental destruction [5]. Some of the embedded pathogens can mount the layers of biofilm and detach from the biofilm membrane in order to migrate and create new biofilm in other places. These bacteria are called “planktonic” and usually are more susceptible to antibiotics than the embedded (called “sessile”) ones [2,3]. The latter expresses biofilm recalcitrance, a problematic mixture of antimicrobial resistance and tolerance. Antibiotic diffusion through the extracellular matrix is problematic, whilst the minimal biofilm inhibitory concentration (MBIC) is many times higher than the minimal inhibitory concentrations (MICs) of both planktonic and non-biofilm bacterial colonies [3].

Moreover, biofilm structures prevent the host from successfully combating them by developing resistance to innate and adaptive immune mechanisms [2,4]. Osteomyelitis and orthopedic implant-associated infections, including fracture-related infections (FRI) and prosthetic joint infections (PJI), are caused by both Gram-positive (GP) and Gram-negative (GN) bacteria able to form biofilms within sequestered bone and/or on the surface of the implant [6]. In order to successfully treat these infections, a combination of surgery and antimicrobial treatment is mandatory [6]. The recommended curative surgery is radical without left debris and it is often followed by the replacement of the infected orthopedic material. Antibiotic treatment options in biofilm-related infections are further limited in the era of emerging resistance [7,8]. No effective eradication therapy is currently available for biofilm-related infections [4]. Therefore, besides surgery, a combination of systemic antibiotics and locally delivered substances with antimicrobial capacities (e.g., antibiotic-loaded cement, antibiotic-coated materials, pre-loaded phages, nanoparticles, quorum-sensing blockers, or monoclonal antibodies) is currently in use or under investigation [2,4]. In the current review, we will assess the preclinical and clinical aspects of an “old” antibiotic, fosfomycin, and its therapeutic role in biofilm-related bone and joint infections (BJI).

### 1.2. Intravenous Fosfomycin

Fosfomycin is a phosphoenolpyruvate analogue that has been available in clinical practice since the 1970s in two oral formulations (i.e., trometamol and calcium) and one intravenous (IV) formulation, FOS disodium (FOS); the interest in the IV formulation has been reawakened in recent years for the treatment of patients with few other treatment options, especially against multi-drug (MDR) GN pathogens [9,10]. FOS is licensed for the treatment of several complicated and deep-seated infections, including BJIs with or without associated bacteremia. The recommended daily dose of FOS for adults with normal renal function is 12–24 g given in 2–4 divided doses, while for pediatric patients (including (preterm) neonates), bodyweight-based dosing is proposed [11].

FOS is a bactericidal antibiotic that acts via the irreversible inhibition of an enzyme-catalyzed reaction in the first committed step of the biosynthesis of the bacterial cell wall at an earlier step than beta-lactams [12]. Due to its unique mechanism of action, cross-resistance to other antibiotic classes is unlikely. FOS is active against both GP and GN bacteria, including MDR pathogens such as methicillin-resistant *S. aureus* (MRSA) and *S. epidermidis* (MRSE), both extended-spectrum beta-lactamase-producing (ESBL) and carbapenem-resistant/carbapenemase-producing (CR/CP) Enterobacterales (including *E. coli* and *K. pneumoniae*); difficult-to-treat (DTR)/CR *P. aeruginosa*; and vancomycin-resistant enterococci (VRE) [13]. Recent data suggest that FOS-containing combination regimens may also be an option in the treatment of *Acinetobacter baumannii* infections [14,15,16,17,18,19]. According to susceptibility data from a surveillance study of more than 2000 randomly selected GP and GN clinical isolates from 109 U.S. medical centers, the MIC required to inhibit the growth of 50% (MIC_50_) and 90% (MIC_90_) of strains was 4 mg/L and 8 mg/L for *S. aureus* (including MRSA), 0.5 mg/L and 2 mg/L for *E. coli*, 4 mg/L and 16 mg/L for *K. pneumoniae*, and 64 mg/L and 128 mg/L for *P. aeruginosa* and *Enterococcus* spp., respectively [20]. Recently, Widerström et al. reported on the in vitro susceptibility of *S. epidermidis* isolates collected from patients with PJIs against FOS. In their study, the MIC_50_ and MIC_90_ of 89 isolates, most of them MDR and/or methicillin-resistant, were 8 mg/L and 32 mg/L, respectively [21].

Regarding antimicrobial susceptibility testing (AST), several methods are available, including automated test systems, gradient tests, agar diffusion, and agar dilution [22,23,24,25], the latter being the reference method recommended by both the European Committee on Antimicrobial Susceptibility Testing (EUCAST) and the Clinical and Laboratory Standards Institute (CLSI). Following a recent revision, the EUCAST currently only provides clinical breakpoints for *E. coli* infections originating from the urinary tract (i.e., 8 mg/L), which applies to situations where FOS is used as a monotherapy [26]. The main reason for revising the clinical breakpoints was that FOS is employed almost exclusively in combination therapy for the majority of pathogens, whereas the clinical breakpoint methodology is predominantly oriented toward monotherapy. For other clinically relevant species (i.e., *Enterococcus* spp.; *Staphylococcus* spp.; *P. aeruginosa*; *Acinetobacter* spp.; and other Enterobacterales, such as *K. pneumoniae*), except for *E. coli* urinary isolates, epidemiological cut-off (ECOFF) values were introduced [27]. As per the EUCAST definition, ECOFFs distinguish isolates without (= wild type (WT)) and with phenotypically detectable acquired resistance mechanisms (= non-WT). ECOFFs (and tentative (T)ECOFFs) can thus be used to monitor acquired resistance. In this context, ECOFFs have also been used as a cut-off to assess the susceptibility of certain pathogens in several studies and in clinical routine, e.g., for enterococci and *P. aeruginosa* [28,29]. Contrary to EUCAST, alternative approaches are recommended in different national AST committees to support the clinical decision to use FOS. While the AST committee in Poland recommends using (T)ECOFFs (i.e., if the measured MIC is ≤species-specific ECOFF, the use of FOS as part of combination therapy is possible) [30], the French CA-SFM retained the former EUCAST breakpoints of 32 mg/L for both Enterobacterales and *Staphylococcus* spp. [31]. From a practical point of view, either of these national recommendations seem reasonable in our opinion, given the important role of AST in decision making in clinical practice.

FOS exhibits synergistic activity with several antibiotic classes, particularly with beta-lactam antibiotics, as demonstrated in several in vitro studies and preclinical models against various GP and GN pathogens, including biofilm involvement. A comprehensive review on these data was published by Antonello and colleagues [32]. Besides its synergistic properties, FOS has also shown activity against intracellular and intraosteoblastic forms of *S. aureus*, which are important in the pathogenesis of BJIs [33,34].

Although FOS alone has been associated with the development of resistance in vitro [35], the emergence of resistance seems to be of minor relevance in clinical practice, as indicated by the results of several studies [10,36,37,38,39], even when used as monotherapy [40,41]. In this context, the results of several in vitro studies have demonstrated that the emergence of resistant subpopulations is suppressed by FOS-containing combination regimens [35,42,43,44,45,46]. Moreover, a study in a rabbit model of infection even showed no development of resistance when FOS was given as monotherapy [47]. In addition, a loss of fitness of resistant strains and the ability of FOS to potentiate bactericidal activity pathways of the immune system may also contribute to the low rates of resistance development in vivo [48,49].

The physiochemical profile of FOS is characterized by a relatively low molecular weight (138 g/mol), negligible plasma protein binding (<5%), and hydrophilic behavior, resulting in good penetration and distribution into several tissues, including soft tissues and bone [50]. FOS elimination occurs almost exclusively via glomerular filtration, with no known metabolism [9]. Following the administration of a single dose of 100 mg/kg of FOS, the concentration achieved in bone and subcutaneous tissue in diabetic patients with severe bacterial foot infections was 43% and 76% of the plasma area under the curve (AUC), respectively. Noteworthy, the concentrations in metatarsal bone and subcutaneous adipose tissue fully equilibrated with plasma levels three hours after infusion [51]. Similar results were found by Legat et al. in patients with cellulitis or diabetic foot infections, where the AUC ratio of subcutaneous soft tissue to plasma was 0.6 to 0.73 [52].

The pharmacodynamic behavior of FOS is still a matter of debate, with some data suggesting that FOS behaves as a time-dependent antibiotic [53,54], while other studies indicate that AUC/MIC or, in the case of *S. aureus*, C_max_/MIC are the most predictive PK/PD (pharmacokinetic/pharmacodynamic) indices [55,56]. Interestingly, one study even found intra-species and inter-species differences in the killing behavior of FOS in ESBL- and/or CP *Enterobacteriaceae*, with some strains appearing to be more time-dependent, while others were concentration-dependent [57]. It should be noted that all these studies evaluated the pharmacodynamics of FOS alone. However, as mentioned above, in clinical practice, FOS is mainly given as part of combination regimens, thus somewhat limiting the relevance of PK/PD data obtained for monotherapy in this clinical scenario. In this context, MacGowan and colleagues recently reported that combining meropenem and FOS had a dramatic impact on both the FOS *f*AUC/MIC and *f*T > MIC exposures required for bacteriostatic and bactericidal effects in an in vitro pharmacokinetic model, providing pivotal data on joint PK/PD [58].

FOS is generally well tolerated, with a very low rate of reported serious adverse events [10]. Due to the sodium content of FOS, electrolyte imbalances, including hypokalemia, are potential adverse drug reactions (ADR) and require monitoring [38]. In this context, the results of a French study indicated that prolonged infusion of FOS may reduce the frequency of hypokalemia associated with its use [59]. Notably, clinical data indicate that FOS has a lower ecological impact on the microbiota of patients than beta-lactams and may even have nephroprotective activity on renal clearance caused by nephrotoxic antibiotics such as aminoglycosides [40,60].

## 2. Materials and Methods

Pubmed/MEDLINE and Google Scholar electronic databases were searched for available literature on in vitro, animal model, and clinical data regarding the use of FOS in biofilm-associated bone and joint infections with a cut-off date of 7 February 2025. Relevant search terms included “fosfomycin”, “biofilm”, “bone and joint infections (including osteomyelitis, osteitis, (septic) arthritis, diabetic foot infection, prosthetic joint infection, (spondylo)discitis)”, “animal (infection) model”, “in vitro”, and “in vivo”. Clinical data were analyzed according to the following parameters: study type, number of patients, type of infection, causative pathogens, daily dose of FOS, antibiotics combined with FOS, duration of FOS treatment, outcome (including clinical and microbiological outcomes), and safety (adverse drug reactions possibly related to FOS therapy).

## 3. Preclinical and Clinical Data

### 3.1. Preclinical Data

Several in vitro and animal model studies have investigated the activity of FOS alone or in combination against biofilm-forming pathogens, including both GP and GN species. Overall, FOS exhibited activity alone, as well as synergistic interactions with various other antimicrobial agents against biofilms. In this context, FOS has been shown to penetrate both newly formed and mature biofilms and even alter their structure [61,62,63]. In the case of *P. aeruginosa*, an increase in the antimicrobial activity of FOS has been described under conditions of limited oxygen availability, resembling those found in biofilm growth [61,64].

While this review focuses on the use of FOS as a treatment option in biofilm-related orthopedic infections, it is noteworthy that FOS has also been investigated as a prophylactic coating or in antibiotic-loaded bone cement, with overall heterogeneous results [65,66,67,68,69,70,71,72,73]. In the following sections, published data on biofilm-related infection models covering important etiological agents of BJIs are discussed.

#### 3.1.1. In Vitro Data

##### *Staphylococcus* spp.

*S. aureus* is a multi-faced bacterial species that can produce toxins but also bears the capacity to exist vicariously in tissues by developing small colony variants (SCVs) or producing biofilms [74]. Compared to concentrations required to inhibit the growth of planktonic cells, FOS generally showed significantly increased MBICs against preformed biofilms of *S. aureus* and *S. epidermidis* [75,76,77,78,79]. However, FOS alone was able to impair the biofilm formation of MSSA and even demonstrated anti-biofilm activity against 24 h MRSA biofilms in vitro [79]. In combination, FOS showed synergistic interactions with several other antibiotics against staphylococcal biofilms, including vancomycin, teicoplanin, rifampicin, cefazoline, and linezolid [74,80,81,82].

##### *Enterococcus* spp.

Several in vitro studies evaluated the activity of FOS alone or in combination with daptomycin against both *E. faecalis* and *E. faecium* biofilms [83,84,85,86]. For example, the results of two studies showed that daptomycin combined with FOS exhibited higher activity against mature biofilms than either daptomycin or FOS alone in linezolid-resistant *E. faecalis* [83,86]. Likewise, FOS with daptomycin showed anti-biofilm activity against VRE isolates [84]. In biofilm-producing vancomycin-resistant *E. faecium* (VREm) isolates, the combination of linezolid plus FOS demonstrated a significant decrease in biofilm biomass and metabolic activity, especially in mature biofilm [87]. In contrast to these findings, antagonistic effects were observed for the combination of ampicillin plus FOS against two of three vancomycin-resistant *E. faecalis* (VREs) isolates tested, and FOS alone did not show anti-biofilm activity against VRE [88]. In another study, FOS alone showed a higher MBC compared to MIC against adherent *E. faecalis* [89].

##### *Escherichia coli* 

Dzib-Baak et al. evaluated the activity of FOS against planktonic and biofilm-forming MDR uropathogenic *E. coli* isolates. In this study, a total of 100 clinical isolates were tested, 83 of which were able to form biofilms, with most being weak or moderate biofilm producers. FOS demonstrated a range of degradative activity against biofilms, with weak producers requiring lower doses of FOS to destroy biofilms compared to moderate and strong producers [90]. In another study, the combination of FOS with gentamicin was highly synergistic (up to 75%) against biofilm-producing *E. coli* strains, even in cases of gentamicin resistance [91]. Likewise, the combination of FOS with either amikacin or meropenem yielded a high percentage of synergy alongside an increased capacity to reduce biofilm formation by MDR *E. coli* [92]. Similar results were observed in the in vitro study conducted by Boncompagni et al., where the combination of FOS plus polymyxin B showed anti-biofilm activity against most *Enterobacteriaceae*, including three MDR *E. coli* strains (two of which co-produced NDM-5 and CTX-M-15) [93].

##### *Klebsiella pneumoniae* 

There is a plethora of studies demonstrating the in vitro synergy of FOS with various antibiotics against planktonic *K. pneumoniae*, including combinations with meropenem, ceftazidime/avibactam, or colistin [32,94,95]. However, data on the anti-biofilm activity against *K. pneumoniae* biofilms are limited. In a study by Ribeiro et al., the combination of FOS plus polymyxin B led to a higher biofilm disruption of contemporaneous KPC-2-producing *K. pneumoniae* clinical isolates compared to both agents alone [96]. In another in vitro study, Ruiz and colleagues observed the anti-biofilm activity of FOS at different concentrations in both microtiter plate assays and endotracheal tubes [97]. Interestingly, low-frequency ultrasound may enhance the activity of antimicrobial agents against *K. pneumoniae* biofilms, including FOS [98]. Contrary to the observed synergistic/additive interactions of ceftazidime/avibactam plus FOS against planktonic *K. pneumoniae*, the combination showed indifferent results against mature biofilms [99].

##### *Pseudomonas aeruginosa* 

In CR biofilm-producing *P. aeruginosa* strains isolated from burn patients, the combinations of FOS with either colistin or gentamicin were effective against planktonic cells, whereas only FOS with colistin, but not FOS plus gentamicin, was effective against biofilm-embedded cells [100]. In a study by Slade-Vitković and colleagues, the in vitro anti-biofilm activity of FOS alone and in combination with other antibiotics against MDR and extensively drug-resistant (XDR) *P. aeruginosa* was investigated. FOS-containing combinations achieved higher rates of biofilm inhibition than single drugs. However, FOS alone did not show an inhibitory effect on the formation of MDR/XDR *P. aeruginosa* biofilms. Notably, no significant effect on the disruption of mature biofilms was observed for either single (e.g., colistin, cefepime, gentamicin, or ciprofloxacin) or combined antibiotics [101]. In another study, the combination of FOS plus tobramycin was synergistic against *P. aeruginosa* strains in two cystic fibrosis biofilm models [102]. Likewise, McCaughey and colleagues observed a bactericidal activity of tobramycin plus FOS against *P. aeruginosa* biofilms under both aerobic and anaerobic growth conditions, while FOS alone was only effective under anaerobic conditions and increased concentrations [103]. A further study demonstrated that the combination of ofloxacin and FOS exhibited higher levels of activity against immature *P. aeruginosa* biofilms than against mature biofilms, with almost no activity observed in the latter. In contrast, single agents demonstrated no activity against biofilms [104]. Synergistic effects of FOS and fluroquinolones against sessile *P. aeruginosa* cells were also observed by Mikuniya and colleagues [105].

##### *Acinetobacter baumannii* 

As mentioned earlier, recent clinical data indicate that FOS-containing regimens may be a valuable option in the treatment of (CR) *A. baumannii* infections [14,15,16,18,106], which is also supported by results of in vitro studies [32,107]. However, experimental data on the in vitro activity of FOS—alone or in combination—against biofilms of *A. baumannii* are limited. In this context, a novel small-molecule non-toxic efflux pump inhibitor potentiates FOS activity against clinical strains of *A. baumannii* and also prevents biofilm formation [108]. In another study, Boncompagni and colleagues evaluated the activity of the combination of FOS and colistin against planktonic cells and biofilms of GN pathogens, including *A. baumannii*. Other species included *P. aeruginosa*, *Stenotrophomonas maltophilia*, *K. pneumoniae*, and *E. coli*. Synergism was observed for the majority of tested strains in both biofilm checkerboard assays (16/20) and quantitative anti-biofilm assays with preformed biofilms (18/20). Of the 20 strains tested in these assays, 3 were CR *A. baumannii* isolates, and synergy was observed for all of them [93].

#### 3.1.2. Animal Models

##### *Staphylococcus* spp.

Most biofilm-related animal model data are available for *S. aureus*. In an experimental implant-associated rat model of MRSA osteomyelitis, FOS was superior to vancomycin in terms of sterilization of bone cultures and implants [109]. Notably, no emergence of resistance was observed in this study. Similar results were observed in another rat MRSA osteomyelitis model, where the combination of vancomycin and FOS showed a synergistic bactericidal effect on biofilm-embedded MRSA, resulting in lower viable colony counts compared to controls and both antimicrobials alone. Interestingly, histological analysis of the pouch wall revealed the disappearance of biofilm structures in animals treated with combination therapy [110]. In a study by Poeppl et al., FOS alone achieved sterilization of 90% of bone cultures, while a combination with daptomycin had no additive effect [111]. Contrary to these findings, results from a chronic implant-associated infection model of MRSA osteomyelitis demonstrated that FOS was highly synergistic if combined with daptomycin [112]. In an MRSA cage-associated infection model, FOS plus rifampicin achieved the highest cure rate (83%) in infected animals compared to other combinations and single drugs. In concordance with previous data, no emergence of FOS resistance was observed [113]. A synergistic effect was also observed in rat models with a combination of FOS and arbekacin or FOS plus linezolid against MRSA biofilms [80,114]. In contrast, in a recently published MRSA vascular graft infection model in rats, the addition of FOS to rifampicin did not increase the efficacy in reducing the bacterial load on grafts compared to FOS alone. In this study, the combination of daptomycin and rifampicin achieved complete sterilization of vascular grafts [115].

##### *Enterococcus* spp.

Oliva et al. investigated the activity of FOS, rifampicin, and various combination regimens against planktonic and adherent *E. faecalis* in an experimental cage-associated animal model of infection. In this study, FOS alone eradicated adherent *E. faecalis* in 43% of cages. In contrast, rifampicin alone had no anti-biofilm activity. The highest cure rate of cages was observed with the combination of FOS plus gentamicin at 58%, while other FOS- or rifampicin-containing regimens eradicated fewer cages than FOS alone [89].

##### *Escherichia coli* 

In an ESBL *E. coli* foreign-body model of infection, the combination of FOS plus colistin showed the highest cure rate (67%) of implanted infected cages compared to other combinations and monotherapies and was the only agent able to eradicate *E. coli* biofilms alone [116]. In an in vivo study by Davido et al., the combination of ceftazidime/avibactam and FOS achieved 100% bone sterilization and significantly decreased bacterial counts in a rabbit model of osteomyelitis caused by OXA-48-/ESBL-producing *E. coli* [117]. Similar results regarding bone sterilization were found in another study by the same group, which evaluated the effect of ceftazidime/avibactam alone or in combination with other drugs, including FOS, colistin, and gentamicin, on the progression of subacute osteomyelitis in rabbits infected with either ESBL/OXA-48-producing *E. coli* or CP *K. pneumoniae*. Overall, all combinations tested were associated with bone sterilization in infected animals (odds ratio = 26.4, 95% confidence interval (CI) [10.2–68.1]; *p* < 0.001) [118]. Noteworthy, FOS, in combination with a new bacteriophage—ϕWL-3—showed high effectiveness in a biofilm larvae model infected with ESBL *E. coli* [119].

##### *Klebsiella pneumoniae* 

In contrast to previous findings, the combination of ceftazidime/avibactam plus FOS did not statistically differ from controls in its efficacy to sterilize bone in an experimental model of KPC *K. pneumoniae* osteomyelitis [120]. In another study, the addition of FOS to colistin in rabbit osteomyelitis due to KPC *K. pneumoniae* effectively reduced bone bacterial concentrations and prevented the emergence of colistin-resistant strains under treatment. However, compared to the triple combination of colistin plus meropenem and gentamicin, colistin combined with FOS did not achieve bone sterilization [121].

##### *Pseudomonas aeruginosa* 

Cai and colleagues evaluated the efficacy of aminoglycosides alone or in combination with FOS against *P. aeruginosa* in a model of biofilm-infected rats. Alone, both FOS and isepamicin had no impact on the reduction in C-reactive protein levels and the number of white blood cells, nor on the reduction in bacterial counts from tissue or silica gel tubes. However, when FOS and isepamicin were combined, viable colony counts were significantly reduced compared to the control group [122]. In a urinary tract animal model of infection, scanning electron microscopy revealed the destruction and disappearance of *P. aeruginosa* multi-layer biofilms on polyethylene tubes in rats after treatment with the combination of prulifloxacin and FOS [123].

### 3.2. Clinical Data

Due to its spectrum of activity and pharmacological properties, FOS is a very attractive option for the treatment of BJIs. Indeed, FOS has been used in clinical practice to treat both pediatric and adult patients with BJIs. Most of the studies published to date are case reports of individual patients, case series, and retrospective observational studies, while no randomized controlled trials (RCT) evaluating the use of FOS for the treatment of BJIs have been conducted so far. Overall, studies are characterized by a heterogeneous patient population and design.

FOS has been used both as first-line or salvage treatment, mainly as part of combination regimens, to treat different types of BJIs caused by various pathogens, including *S. aureus*, enterococci, Enterobacterales, and *P. aeruginosa*. According to a recent review by Tsegka et al., covering studies published up to 2020 (including data from 19 published studies), the overall treatment success rate in a total of 365 patients was favorable, at 82.2%, and the drug appeared to be generally well tolerated. Adverse events included cutaneous allergic reactions and mild gastrointestinal discomfort as the most frequent; hematological side effects (neutropenia and leukopenia) were rare, as were electrolyte imbalances [124]. A summary of available clinical data is provided in Table 1, and important studies are discussed in more detail in the following section.

### 3.3. Studies in Adults

In a study from the late 1980s, Meissner and colleagues published the results of a prospective study on 60 adult patients with chronic post-traumatic osteomyelitis caused by various pathogens, including *S. aureus* (42%), CoNS (19%), *P. aeruginosa* (12%), streptococci (7%), and enterococci (5%). FOS as monotherapy was given in a daily dose of 15 g as a second-line option and started immediately before surgery, then continued for a minimum of five to a maximum of 28 days (with a mean duration of 13.9 days). Prior to FOS therapy, patients had a mean history of disease of 37 months, had been treated previously with up to 12 (mean 3.1) antibiotic courses, and had, on average, 2.4 operations. At follow-up examination, the outcome was classified as positive in the majority of patients (73.6%), while a relapse occurred in 26.4%. FOS was generally well tolerated, with only mild adverse effects during therapy, including gastrointestinal disorders, phlebitis, and allergic exanthema [128].

In 2005, Stengel and colleagues reported on 52 patients (mean age of 62.9 years) with limb-threatening diabetic foot infections (i.e., Wagner grade 3 and higher) from five study centers in Austria receiving FOS treatment within a compassionate use program. In around half of the patients, antibiotic pre-treatment had failed. The majority of patients had infections caused by *S. aureus*, *Streptococcus* spp., and *Proteus* spp. Other pathogens mainly involved GN, including *Pseudomonas* spp., *E. coli*, and *Citrobacter freundii*. In seven patients, diabetic foot infections were caused by enterococcal species. Patients included were treated for a mean duration of 14.4 days with FOS, ranging from 3 to 40 days. The mean daily dose of FOS was 14.9 g, and most patients received FOS as part of a combination regimen (82.7%). Partner antibiotics included beta-lactams, ciprofloxacin, and clindamycin. Overall, the affected limb could be salvaged in 48 of the 52 patients (92.3%). Clinical cure was achieved in 25% and marked improvement in 59.6% of patients, respectively. Treatment failure occurred in eight patients. Adverse events were negligible (four cases of mild nausea and rash, and no cases of serious adverse events), and FOS was generally well tolerated [131].

Dinh et al. reported on the clinical use of FOS in France during a crisis in drug production; during this 10-week shortage period, all prescriptions were strictly monitored. Among 101 patients who received FOS, all in combination with other antibiotics, 32 had BJIs caused by both GP and GN pathogens. In 41 patients, a foreign body material (i.e., orthopedic implant or catheter) was present. In the subgroup of patients with BJIs, FOS was given for a mean of 49.3 days, and a favorable outcome was reported in 82.6% of clinically evaluable patients (19/23) [134].

More recent data were published by Putensen et al. describing a cohort of 209 patients treated with FOS in 20 intensive care units (ICUs) in Germany and Austria, including 21 cases of BJIs (± bacteremia/sepsis). In accordance with other studies, FOS was mainly used as part of combination therapy (99%) in this prospective multicenter study. In the clinically evaluable population, clinical success was achieved in 81% (148/182) of patients, while clinical success and microbiological eradication in patients treated for BJIs were 85.7% (18/21) and 100% (13/13), respectively. Noteworthy, no emergence of resistance under FOS therapy was observed. A total of 39 patients in the intent-to-treat population developed at least one ADR, including hypokalemia (2.5%) and hypernatremia (10.5%). However, treatment-related adverse effects were, in most cases, non-serious [38].

Anastasia et al. published the results of a retrospective monocentric study conducted in Italy covering the period between 2017 and 2022. In total, 343 adult patients were treated with FOS for various types of infections, including 37 cases of osteomyelitis. Infections were mainly caused by GN pathogens. FOS was given in a dose of 16 to 24 g/d, most often in combination with other antimicrobial agents (324/343; 94.4%). Companion antibiotics included ceftazidime/avibactam, meropenem, colistin, vancomycin, and daptomycin. Overall, clinical recovery at the end of treatment was achieved in 66%, and adverse events occurred in 5.8% (20/343) of patients, including four cases each of hypernatraemia (1.2%) and hypokalemia (1.2%). Compared to the overall cohort, the clinical outcome was more favorable in patients treated for osteomyelitis (recovery in 31/37; 83.7%). Specific data on adverse events were not provided for the subgroup of osteomyelitis patients [39].

Very recently, Meschiari et al. published the results of a cohort of patients with DTR infections caused by MDR GN bacteria treated with FOS. A total of 12 of the 70 patients included in this retrospective observational study were treated for osteomyelitis/PJI. The median Charlson comorbidity index was 4 (interquartile range of 3–6) in the total population, and every third patient was concomitantly diagnosed with COVID-19. *P. aeruginosa*, *K. pneumoniae*, and *E. coli* were the most frequently isolated pathogens. Overall, a significant proportion of isolates exhibited resistance to carbapenems, ceftazidime/avibactam, and ceftolozane/tazobactam (54.3%, 31.4%, and 27.8%, respectively). FOS was given in a range of 16–24 g/day and was most often combined with ceftazidime/avibactam, meropenem, or cefiderocol. In the subset of patients suffering from BJIs, a clinical cure and microbiological cure were achieved in 75% (9/12) and 83.3% (10/12), respectively. While the overall all-cause mortality rate at day 90 was 31.4%, none of the patients in the osteomyelitis/PJI subgroup died [162].

More specific data on PJIs come from a retrospective study by Renz et al. describing a cohort of 75 consecutive patients with enterococcal PJIs (both *E. faecalis* and *E. faecium*, most of the isolates being vancomycin-susceptible) [28]. Patients were treated with either a penicillin derivative, vancomycin, or daptomycin as monotherapy or in combination with an adjunctive agent; FOS was prescribed as a combination partner in 25 cases. About half of the patients had polymicrobial infections, mostly caused by CoNS or GN bacilli. The median total duration of antibiotic treatment was 16 weeks, while the median duration of FOS treatment was 14 days. Outcome data were available for 88% of patients (66/75) with follow-up data and completed antibiotic therapy. Overall, treatment success (i.e., relapse-free probability) after 3 years was achieved in 83.7%. Although there was no statistical difference between patients receiving combination or monotherapy (88% vs. 73%, *p* = 0.217), patients treated with FOS-containing regimens had numerically better outcomes (95%) than those without FOS (80%) [28].

Interim results from two currently ongoing prospective multicenter studies, PROOF and FORTRESS (NCT02979951), have shown promising results. The PROOF study evaluates the efficacy and safety of FOS-based regimens in a pathogen/surgery-specific mode according to a standardized algorithm for the treatment of PJIs, including step-down therapy with oral antibiotics. PJIs were most frequently caused by CoNS and MSSA. The infection-free rate after 2 years was 85% (analyzed for 164 patients). ADRs reported included nausea, diarrhea, and electrolyte imbalances [143]. In addition, Bodmann and colleagues very recently published interim results of the FORTRESS study. In concordance with other studies, FOS was almost exclusively used in combination therapy. The clinical response and microbiological cure rates in patients with different types of BJI were 79.8% and 81.5%, respectively, while specific safety data for this subgroup were not reported [146].

Besides case series and observational studies, several case reports on the use of FOS in DTR biofilm-related orthopedic infections have been published. For example, Wong et al. and Narayanasamy et al. reported on the successful use of FOS in combination therapy in the treatment of MDR/XDR *P. aeruginosa* BJI cases [139,142].

### 3.4. Studies in Pediatric Patients

In 1989, Guggenbichler and colleagues described a cohort of 36 pediatric patients with acute hematogenous osteomyelitis treated with a combination of FOS (250 mg/kg/d bodyweight, divided into three doses) plus either oxacillin or cefamandole for 10–14 days, followed by oral clindamycin for 3–6 weeks. Osteomyelitis was most frequently due to *S. aureus*. However, about half of the cases were culture-negative. Overall, 34 of 36 were successfully treated with this treatment regimen [149].

A comparative study was published in 2003 by Corti et al. The study compared 103 children with acute hematogenous osteomyelitis who were initially treated conservatively (without surgery) with FOS alone (N = 23), FOS plus other antimicrobials (N = 47), or other antimicrobials alone (including flucloxacillin, amoxicillin, amoxicillin/clavulanic acid, and clindamycin) (N = 33). Staphylococci and streptococci were the most frequently isolated bacterial species. However, in the majority of patients, no causative agent could be identified. Overall, similar efficacy was observed between the three treatment groups [153].

In a very recent study, medical records of 20 pediatric patients with a mean age of 10.2 years from an Italian hospital were analyzed. Most of the patients presented with osteoarticular infections (65%; 13/20). Other infections included endocarditis, pneumonia, and bacteremia. On average, patients were treated for 18 days. Despite the complicated nature of these infections, all patients were successfully treated with FOS-containing regimens and discharged in good clinical condition. Mild ADRs were observed in a total of eight patients [156].

## 4. Discussion

BJIs are considered difficult-to-treat infections due to the deep-seated site of infection and the role of biofilms in pathogenesis, regardless of the presence of foreign body materials. Together with adequate surgical measures, the use of antibiotics with established anti-biofilm activity is crucial for the successful treatment of such infections. FOS has emerged as a promising option in this context, demonstrating activity alone and in combination with other antibiotics against both GP and GN pathogens in in vitro and animal models of biofilm-related infections [32,90,93,96,112,117,118]. This characteristic, together with good and fast bone penetration at therapeutic doses [51], represents a strong theoretical premise to support the clinical use of FOS in treating BJIs. Notably, FOS is approved for the treatment of BJIs [11].

Analysis of the published literature on the use of FOS in BJIs revealed that the drug was initially employed predominantly as an anti-staphylococcal agent [128,131,153,161], while more recently, it has also been gaining a role as part of combination regimens for the treatment of biofilm-related orthopedic infections caused by MDR GN pathogens [134,139,142,145]. In this context, the treatment of BJIs caused by MDR GN bacteria is hampered by the inability to use fluoroquinolones, which, due to their anti-biofilm activity and good penetration into bone tissue, are the cornerstone of the treatment of susceptible GN BJIs [163,164,165].

Overall, the treatment of various types of BJIs with FOS-containing regimens—predominantly in combination therapy—resulted in favorable clinical and microbiological outcomes in both adult and pediatric populations, including even neonates and complex/challenging cases, with success rates often exceeding 80%. Due to the larger number of studies, including infections caused by GP pathogens (predominantly due to staphylococci), data are more robust for the use of FOS in GP BJIs as opposed to BJIs due to (MDR) GN bacteria. In general, FOS appeared to be well tolerated in most studies and patient populations, with only a few ADRs reported. This is an important finding as the treatment of BJIs usually requires prolonged treatment courses (at least 4 weeks according to current recommendations [166,167,168]), and drug tolerability is crucial. Indeed, high rates (35%) of unplanned treatment discontinuations due to adverse events were reported in patients receiving fluoroquinolones for the treatment of PJIs [169], and the rate of adverse events linked to rifampicin, another drug of choice for biofilm-associated infections, in PJI varies between 4.3% and 31.2% [170,171,172].

However, when interpreting the available clinical data, some limitations should be considered. Firstly, most of the clinical data derive from retrospective observational studies, case reports, and case series rather than prospective clinical studies. Secondly, comparing studies is challenging due to the differences in patient population, etiologies, infection types, dosage and combination regimens, and definitions of clinical/treatment success. Furthermore, sequential oral antibiotic therapy was used in some studies, which should be taken into account when evaluating particularly long-term outcomes. Another aspect to be considered is that some studies were conducted before the advent of good clinical practice. Finally, publication bias (i.e., published case reports and case series usually refer to successful clinical experience) could have led to an overestimation of the efficacy of the drug.

Despite the lack of RCTs and limited prospective data, it should be emphasized that the value of real-world data is increasingly recognized to provide information about the effectiveness of drugs in heterogeneous patient populations, including individuals who cannot be enrolled in RCTs. Indeed, patients participating in clinical trials are selected according to certain inclusion and exclusion criteria and may differ significantly from those encountered in a real-life setting [173], especially with respect to comorbidities and criticality of illness, older age, and the use of concomitant medications. The forthcoming results from two large prospective studies, FORTRESS and PROOF, are expected to provide more robust data on the use of FOS in various BJIs, particularly PJIs.

## 5. Conclusions

Based on published literature, preclinical and clinical data, although heterogeneous, support the use of FOS as part of combination regimens for the treatment of different BJIs across all age groups. Given its activity and anti-biofilm properties—especially in combination—against both GP and GN agents, FOS may be used empirically or as targeted first-line treatment but may also offer an option in difficult-to-treat cases where the initial antibiotic therapy has failed. In addition, its excellent penetration into bone tissue, its unique mode of action and intracellular activity, and its favorable safety profile make FOS a reasonable choice for the treatment of biofilm-related BJIs.

While most of the studies reported on the clinical effectiveness of FOS in BJI caused by GP agents, FOS appears to also be attractive for the treatment of biofilm-related orthopedic infections caused by MDR/XDR GN bacteria, for which only a limited arsenal of active substances is available. For BJIs due to methicillin-resistant *Staphylococcus* spp. or enterococci, the combination of FOS plus daptomycin appears to be promising based on the results of in vitro and animal models [84,85,86,112], with an FOS dose of up to 16 g. For the treatment of BJI caused by GN bacteria such as CR *K. pneumoniae* or MDR *P. aeruginosa*, higher FOS doses (up to 24 g) may be used, and FOS should be combined with an adequate antibiotic backbone, including novel beta-lactam/beta-lactamase inhibitors (BL/BLI) such as ceftazidime/avibactam. In this context, the successful treatment of a patient with osteomyelitis due to MDR *P. aeruginosa* using a combination of ceftazidime/avibactam and FOS was reported by Mancuso et al. [174]. Given that hypokalemia and hypernatremia are possible ADRs, electrolytes should be regularly monitored, particularly when elevated daily doses are administered.

To address the limitations of current evidence, future efforts should be directed at conducting well-designed prospective studies, including comparative clinical trials to define the role of FOS-containing regimens relative to standard-of-care therapies. Furthermore, these studies could help optimize combination regimens, FOS dosing (including further investigations on continuous infusion [175]), and treatment duration for different BJIs and further evaluate potential adverse events associated with FOS use. Multicenter clinical studies are warranted to close these gaps, minimize bias introduced by the predominantly retrospective nature of available clinical data, confirm the observed favorable outcomes, and further refine the role of FOS in treating complex biofilm-associated BJIs.

## Figures and Tables

**Table 1:** Summary of clinical data on the use of IV fosfomycin for the treatment of bone and joint infections.

First Author	Study Type	Patients [N] (Age) *	Type of Infection	Causative Agents	FOS Daily Dose	Combination Partner(s)	FOS Treatment Duration	Outcome	Safety **
*Adult patients*									
Yoh [125]	Case series	8 (range: 17–53 y)	Osteomyelitis	Miscellaneous	8 g	FOS monotherapy	7 d (mean)	4/8 “Good” (50%) 4/8 “Fair” (50%)	No ADRs
Baron [126]	Retrospective study	20 (mean: 39 y in total population (N = 105))	Acute osteomyelitis (N = 12), arthritis (N = 8)	*Staphylococcus* spp.	200 mg/kg	Oxacillin (N = 17), pefloxacin (N = 2), aminoglycoside (N = 1)	NA	10/20 cured (50%), 9/20 improved (45%), 1/20 failure (5%)	Hypokalemia (N = 10), hypokalemia plus hypernatremia (N = 6), rash (N = 3)
Watanabe [127]	Case report	1 (82 y)	Purulent arthritis (knee joint)	MRSA	8 g	Minocycline and cloxacillin	NA	Cured	NA
Meissner [128]	Prospective study	60 (mean: 37.4 y)	Chronic post-traumatic osteomyelitis	*S. aureus* (N = 34), CoNS (N = 15), *P. aeruginosa*, streptococci (N = 10), GN aerobes (N = 19, including *P. aeruginosa* and Enterobacterales), anaerobic bacteria (N = 1)	15 g	FOS monotherapy	5–28 d (range)	39/60 favorable outcome (i.e., “very good”, “good”, or “satisfactory”; 73.6%), 14/60 relapse (26.4%); 7/60 unevaluable (i.e., lost to follow-up; 11.7%)	Exanthema (N = 2), GI disorders (mild) (N = 4), phlebitis (N = 7)
Lucht [129]	Open-label prospective study	2 (21 y and 26 y)	Chronic osteomyelitis	*P. aeruginosa*	12 g	Cefsulodine (N = 1), amoxicillin plus ciprofloxacin (N = 1)	5.5 m	2/2 cured (100%)	No ADRs observed during FOS therapy
Bureau-Chalot [130]	Case report	1 (43 y)	Post-operative pyogenic spondylodiscitis	*Stomatococcus mucilaginosus*	12 g	Cefotaxime	2 w	Cured	NA
Stengel [131]	Prospective study	52 (mean: 62.9 y)	Limb-threatening diabetic foot osteomyelitis	*S. aureus* (N = 24), *Streptococcus* spp. (N = 14), *Enterococcus* spp. (N = 7), GN bacteria (N = 27)	8–24 g	Meropenem (N = 14), amoxicillin + BLI (N = 12), clindamycin (N = 10), ciprofloxacin (N = 10), ceftriaxone (N = 4), imipenem (N = 2), others (N = 5)	14.4 d (mean; range: 3–40 d)	13/52 clinical cure (25%); 31/52 marked improvement (59.6%); 8/52 treatment failure (15.4%)	Nausea and rash (mild) (N = 4)
Gillard [132]	Case series	3 (range: 53–67 y)	Pyogenic discitis	Culture-negative	NA	Fluoroquinolone (N = 2), cephalosporin (N = 1)	18.3 days (mean)	3/3 cured (100%)	NA
Izumi [133]	Case report	1 (73 y)	Vertebral osteomyelitis	*Streptococcus pneumoniae*	4 g	Panipenem/betamipron	2 w	Cured	NA
Dinh [134]	Prospective cohort study	32 (NA)	BJI	CoNS (N = 10), *Klebsiella* spp. (N = 7), *E. coli* (N = 6), *P. aeruginosa* (N = 5), MRSA (N = 4), *Streptococcus* spp., MSSA (N = 3), *Aeorococcus viridans* (N = 1), *Citrobacter* spp. (N = 1)	12–16 g	Glycopeptide (N = 10), cephalosporin (N = 9), carbapenem (N = 8), fluoroquinolone (N = 4), methicillin (N = 2), rifampicin (N = 3), metronidazole (N = 1), cefepime (N = 1), colistin (N = 1)	54 d (mean)	19/32 favorable (59.4%), 4/32 unfavorable (12.5%), 1/32 early death (3.1%), 8/32 insufficient follow-up (25%)	Hypovolemia (N = 3), neutropenia (N = 1)
Lee [135]	Case report	1 (85 y)	Vertebral osteomyelitis	MRSA	16 g	Teicoplanin	5 w	Cured	NA
Luengo [136]	Case report	1 (79 y)	Total femoral replacement infection	MDR *S. epidermidis*	8 g	Daptomycin	42 d	Cured	No ADRs
Baron [137]	Case report	1 (43 y)	FRI	OXA-48/NDM-producing *K. pneumoniae*	12 g	Colistin and doxycycline	3 m	Cured	No ADRs
Putensen [38]	Prospective, non-interventional multicenter study	21 (mean: 59.1 y in total population (N = 209))	BJI	NA	13.7 g (mean; in total population (N = 209))	NA	20 d (mean)	18/21 clinical success (85.7%)	NA
Renz [28]	Retrospective cohort study	25 (median: 76 y in total population (N = 75))	PJI	*Enterococcus* spp. (most patients in the total population (N = 75) had *E. faecalis* PJI (85%); 50.7% of PJIs were polymicrobial)	15 g	Penicillin G, ampicillin, vancomycin, daptomycin, gentamicin	14 d (median; range: 3–90 d)	95% treatment success at 3-year follow-up in patients treated with FOS-containing regimens	NA
Rieg [138]	Prospective observational cohort study (post hoc analysis)	37 (median: 67 y in patients treated with combination therapy (N = 313))	Bacteremic osteoarticular infections	*S. aureus*	15 g	NA	14 d (median; in all patients treated with FOS (N = 58))	27% mortality (in 165 patients with bacteremic osteoarticular infections treated with combination therapy); FOS combination therapy vs. monotherapy in this subgroup: 90-day mortality (HR 0.68, 95% CI [0.24–1.91], *p* = 0.460]; death or SAB-related complications (HR 0.71, 95% CI [0.27–1.88], *p* = 0.496)	NA
Narayanasamy [139]	Case report	1 (75 y)	FRI	XDR *P. aeruginosa*	16 g	Colistin	12 w	Cured	No ADRs
Nakamura [140]	Case report	1 (84 y)	Vertebral osteomyelitis (plus bilateral psoas abscess)	MRSA	4 g	Imipenem/cilastatin	4 w	Clinical improvement	NA
Kehila [141]	Case report	1 (34 y)	Post-partum pubic symphysite	Group B *Streptococcus*	NA	3rd-generation cephalosporin	8 w	Cured	NA
Wong [142]	Case report	1 (84 y)	Osteomyelitis	MDR *P. aeruginosa*	18 g	Ceftolozane/tazobactam and meropenem	2 w	Cured	Hypokalemia
Karbysheva [143]	Prospective, interventional, investigator-initiated multicenter study	168 (median: 74 y; range: 18–88 y)	PJI	MSSA (N = 28), CoNS (N = 58), *Enterococcus* spp. (N = 15), *Streptococcus* spp. (N = 13), GN rods (N = 14), other (N = 14), culture-negative (N = 40)	15 g	NA	NA	85% (infection-free rate after 2 years)	Hypokalemia (N = 49), nausea (N = 56), diarrhea (N = 10), hypernatremia (N = 13)
Pignatti [144]	Case report	1 (49 y)	Sternal osteomyelitis	*Klebsiella aerogenes*	NA	Ertapenem	3 w	Cured	NA
Anastasia [39]	Retrospective observational study	37 (mean: 68 y in total population (N = 343))	Osteomyelitis	NA	16–24 g	NA	NA	31/37 recovery (83.8%), 2/37 relapse (5.4%), 2/37 death (5.4%)	NA
Meschiari [145]	Retrospective observational study	12 (median: 69 y in total population (N = 70))	Osteomyelitis, PJI	MDR GN bacteria	16–24 g	NA	NA	9/12 clinical cure (75%), 10/12 microbiological cure (83.33%), 0/12 30-day all-cause mortality	NA
Bodmann [146]	Prospective, non-interventional multicenter study	124 (mean: 62.8 y in total population (N = 716))	BJI	NA	15 g (median; in total population (N = 716)	NA	NA	90/124 clinical response (79.8%), 101/124 microbiological cure (81.5%)	NA
*Pediatric patients*									
Gouyon [147]	Case series	2 (24 d)	Osteomyelitis	*S. aureus*	50 mg/kg	Cefotaxime	14 d	2/2 cured (100%)	Hypernatremia (N = 2)
Badelon [148]	Prospective study	20 (mean: 3.5 y in total population (N = 23))	Osteomyelitis (N = 8) and arthritis (N = 12)	*S. aureus,* *Haemophilus influenzae*	105 mg/kg	Cefotaxime	15 d	20/20 cured (100%)	No ADRs
Guggenbichler [149]	Prospective study	36 (NA)	Acute hematogenous osteomyelitis	*S. aureus* (N = 16), other bacteria (N = 4), culture-negative (N = 15)	250 mg/kg	Cefamandol or oxacillin	10.5 d (mean; range: 10–14 d)	34/36 successful outcome (94.4%)	Neutropenia (n = 1)
Stricker [150]	Case report	1 (5 y)	Osteomyelitis and septic arthritis	*H. influenzae* and *C. freundii*	200 mg/kg	FOS monotherapy	4 w	Cured	NA
Briard [151]	Case report	1 (2 y)	Acute osteomyelitis (plus necrotizing fasciitis)	*Streptococcus* spp. (beta-hemolytic group A)	100 mg/kg	Cefotaxim	30 d	Cured	NA
Reinehr [152]	Case series	10 (range: 8–16 y)	Chronic osteomyelitis	NA	200 mg/kg	Penicillin G	21 d (mean)	9/10 recovery (90%), 1/10 relapse (10%)	NA
Corti [153]	Retrospective study	70 (Group 1: mean age 6.0 y; group 2: mean age 7.0 y)	Acute hematogenous osteomyelitis	*S. aureus* (N = 15), CoNS (N = 6), *Streptococcus pyogenes* (N = 2), *S. pneumoniae* (N = 1)	200 mg/kg	Group 1: FOS alone (N = 23), Group 2: FOS in combination (N = 47; including flucloxacillin (N = 38), clindamycin (N = 2), amoxicillin (N = 2), amoxicillin/clavulanic acid (N = 4), gentamicin (N = 1))	Group 1: 2.5 w (mean), Group 2: 3.1 w (mean)	70/70 recovery (100%)	Diarrhea (N = 2), exanthema (N = 10), leucopenia (N = 1)
Fitoussi [154]	Case series	18 (mean: 6.5 y; range: 9 m–14 y)	Hematogenous wrist osteomyelitis	MSSA (N = 7), MRSA (N = 1)	NA	Cefotaxime	7 d IV therapy, 6 w total antibiotic therapy	15/18 cured (83.3%)	NA
Allagui [155]	Case report	1 (30 d)	Acute osteomyelitis	*H. influenzae*	NA	Cefotaxime	3 w	Cured	NA
Roversi [156]	Case series	13 (mean: 10.2 y in total population (N = 20))	osteomyelitis (N = 10), arthritis (N = 3)	*Fusobacterium necrophorum* (N = 1), *Prevotella oris* (N = 1), MSSA (N = 1), *E. coli* (N = 1), *Streptococcus oralis* (N = 1), MRSA (N = 1), Staphylococcus *cohnii* (N = 1), culture-negative (N = 6)	12 g (>40 kg), 400 mg/kg (10–40 kg), 200 mg/kg (ges-tational age < 40 w)	Meropenem (N = 4), ciprofloxacin (N = 1), ceftriaxone (N = 5), linezolid (N = 2), teicoplanin (N = 2), rifampicin (N = 1), vancomycin (N = 1)	18.4 d (mean)	13/13 recovery (100%)	Fatigue (N = 1), anorexia (N = 1), phlebitis (N = 2), tachycardia (N = 1), nausea/vomiting (1), hyper-transaminasemia (N = 3)
*Miscellaneous*									
Fernandez-Valencia [157]	Case series	37 (range: 4–75 y)	Osteomyelitis	*S. aureus*	4–8 g	FOS monotherapy (oral route (N = 5), intramuscular (IM) route (N = 13), oral plus IM route (N = 19)	3 w (mean)	29/37 cured after 3–4 years of follow-up (78.4%), 35/37 initial success (94.6%)	No ADRs were observed during IV FOS therapy (2 patients switched from IM to IV route)
Hernandez-Casado [158]	Case series	3 (range: 7–74 y in total population (N = 99))	Osteomyelitis	*S. aureus*	8–16 g	FOS monotherapy	2–4 d (range)	3/3 cured (100%)	NA
Portier [159]	Prospective study	10 (range: 2.5 m–71 y)	BJI	*S. aureus* (including 2 cases with MRSA involvement)	150 mg/kg	Cefotaxime	16.5 d (mean)	10/10 cured (100%)	Cerebral edema (N = 1)
Portier [160]	Prospective study	6 (range: 4–69 y)	BJI	MRSA (N = 6), *Alcaligenes faecalis* (N = 1), group D *Streptococcus*	150–200 mg/kg	Cefotaxime	11–21 d (range)	6/6 (100%)	No ADRs
Stöckl [161]	Retrospective observational study	40 (median: 60 y; range: 14–80 y)	Spondylodiscitis	*S. aureus* (N = 21), *Streptococcus* spp. (N = 3), *E. coli* (N = 3), *S. epidermidis* (N = 2), *Enterococcus* spp. (N = 1), *Salmonella* spp. (N = 1), culture-negative (N = 11)	8–24 g	Cephalosporin (N = 22), other beta-lactams (N = 5), clindamycin (N = 7), rifampicin (N = 6), vancomycin (N = 3), metronidazole (N = 1)	24 d (median; range: 3–89 d)	35/40 clinical success (87.5%; 30/40 cured, 5/40 improved), 5/40 failure (12.5%)	Flushing and taste disturbance (N = 1)

*—Treated with FOS for BJI; **—only adverse drug reactions possibly related to FOS treatment are listed; *ADR*—adverse drug reaction; *ALT*—alanine transaminase; *BJI*—bone and joint infection; *CI*—confidence interval; *CoNS*—coagulase-negative staphylococci; *d*—day(s); *FOS*—fosfomycin; *g*—grams; *FRI*—fracture-related infection; *GI*—gastrointestinal; *GN*—Gram-negative; *HR*—hazard ratio; *IM*—intramuscular; *IV*—intravenous; *m*—month(s); *MDR*—multidrug-resistant; *MRSA*—methicillin-resistant *S. aureus*; *MSSA*—methicillin-susceptible *S aureus*; *NA*—not applicable (either not reported or not observed); *NDM*—New Delhi metallo-beta-lactamase; *OXA*—oxacillinase; *PJI*—prosthetic joint infection; SAB—*S. aureus* bacteremia; *XDR*—extensively drug-resistant; *y*—year(s).

## Data Availability

No new data were created or analyzed in this study. Data sharing is not applicable to this article.

## Abbreviations

ADR	Adverse drug reaction.
ALT	Alanine transaminase.
AST	Antimicrobial susceptibility testing.
AUC	Area under the curve.
BJI	Bone and joint infection.
BL/BLI	Beta-lactam/beta-lactamase inhibitor.
CI	Confidence interval.
CLSI	Clinical and Laboratory Standards Institute.
C_max_	Maximum serum concentration.
CoNS	Coagulase-negative staphylococci.
COVID-19	Coronavirus disease 2019.
CP	Carbapenemase-producing.
CR	Carbapenem-resistant.
CTX-M	Cefotaximase-Munich.
EPS	Extracellular polymeric substances.
ESBL	Extended-spectrum beta-lactamase.
EUCAST	European Committee on Antimicrobial Susceptibility Testing.
FOS	Fosfomycin disodium.
FRI	Fracture-related infection.
GI	Gastrointestinal.
GN	Gram-negative.
GP	Gram-positive.
HR	Hazard ratio.
ICU	Intensive care unit.
IM	Intramuscular.
IV	Intravenous.
KPC	*K. pneumoniae* carbapenemase.
MBIC	Minimal biofilm inhibitory concentration.
MDR	Multidrug-resistant.
MIC	Minimal inhibitory concentration
MRSA	Methicillin-resistant *Staphylococcus aureus.*
MRSE	Methicillin-resistant *Staphylococcus epidermidis.*
MSSA	Methicillin-susceptible *Staphylococcus aureus.*
NA	Not applicable.
NDM	New Delhi metallo-beta-lactamase.
OXA	Oxacillinase.
PK/PD	Pharmacokinetic/pharmacodynamic.
PJI	Prosthetic joint infection.
SAB	Staphylococcus aureus bacteremia.
SCV	Small colony forming variant.
(T)ECOFF	(Tentative) Epidemiological cut-off.
T > MIC	Time above MIC.
VRE	Vancomycin-resistant enterococci.
VREm	Vancomycin-resistant *Enterococcus faecium.*
VREs	Vancomycin-resistant *Enterococcus faecalis.*
WT	Wild type.
XDR	Extensively drug-resistant.
